# Construction and Validation of a Ferroptosis-Related Prognostic Signature for Melanoma Based on Single-Cell RNA Sequencing

**DOI:** 10.3389/fcell.2022.818457

**Published:** 2022-03-03

**Authors:** Yating Liu, Yanhong Shou, Ronghui Zhu, Zhuoqiong Qiu, Qi Zhang, Jinhua Xu

**Affiliations:** ^1^ Department of Dermatology, Huashan Hospital, Fudan University, Shanghai, China; ^2^ Shanghai Institute of Dermatology, Shanghai, China

**Keywords:** ferroptosis, prognosis, melanoma, single cell, gene signature

## Abstract

Melanoma, the deadliest type of skin cancer, is on the rise globally. The generally poor prognosis makes melanoma still an enormous public health problem. Ferroptosis is a newly emerging form of iron-dependent regulated cell death, which has been implicated in the development and treatment of several tumors. However, whether there is a connection between ferroptosis-related genes and the prognosis of melanoma patients remains an enigma. In the present study, we identified a ferroptosis-related genes signature to predict the prognosis of melanoma patients by analyzing single-cell RNA-sequencing data from the Gene Expression Omnibus (GEO). Single-cell trajectory analysis was performed to explore malignant differentiation. CellChat was used to investigate intercellular communications in melanoma. Collectively, a novel four-gene signature (CP, MAP1LC3A, transferrin, and TP53) was constructed for prognosis prediction. COX proportional hazards regression analysis showed that the established ferroptosis-associated risk model was an independent prognostic predictor for melanoma patients (HR = 2.3293; 95%CI 1.1528–4.706) (*p* < 0.018). Patients with low-risk scores had significantly better overall survival (OS) than those with high-risk scores in The Cancer Genome Atlas, GSE59455, and GSE22153 dataset (*p* = 0.0015, *p* = 0.031, *p* = 0.077). Furthermore, the gene expression level of the four genes were verified in multistrain melanoma cell lines and normal human epidermal melanocytes (NHEM). The protein expression level of the four genes in clinical samples were further verified in the Human Protein Atlas (HPA) databases. Taken together, our study identified the prognostic significance of the ferroptosis-related genes in melanoma and developed a novel four-gene prognostic signature, which may shed light on the prognostic assessment and clinical decision making for melanoma patients.

## Introduction

Melanoma is the most aggressive and fatal malignant cutaneous neoplasm which arises from a malignant transformation of melanocytes (Schadendorf et al., 2018; Fisher, 2021; Jenkins and Fisher, 2021). In the past few decades, the incidence of melanoma has risen dramatically worldwide (Leonardi et al., 2018; Mishra et al., 2018). Thought melanoma accounts for one percent of all skin cancers diagnosed, it causes the vast majority (75%) of deaths associated with skin cancer due to its strong metastasis ability and poor prognosis (Schadendorf et al., 2015; Weiss et al., 2019). The modern treatment landscape for melanoma includes surgery, chemotherapy, immunotherapy, or targeted therapy which is used alone or combined therapies depending on the features of the tumor (Lee et al., 2021). There is no doubt that the treatment of melanoma has made unprecedented progress in the past decade (Domingues et al., 2018). Unfortunately, resistance to available treatment modalities and recurrence of melanoma often occur so that the prognosis of melanoma is still not optimistic (Ding et al., 2019). Moreover, there are several side effects associated with these therapies as they also harm normal cells during the treatment process (Lee et al., 2021). Therefore, it is of great value to investigate novel and effective prognostic signature as new therapeutic strategies with fewer side effects for melanoma, so as to provide precision medical service and obtain better clinical benefit.

Cell death is an indispensable part of life with far-reaching implications in diverse aspects of multicellular organisms development, homeostasis, and disease (Gao and Jiang, 2018). Regulated cell death (RCD) is controlled by specific signal transduction pathways, making them eligible to be modulated pharmacologically or genetically (Chen et al., 2021). Almost all existing cancer treatment strategies are designed to selectively kill cancer cells (Chen et al., 2021). Considerable efforts have been made to use RCD in cancer therapy (Liang et al., 2019). As the most famous and best studied RCD, apoptosis is the key target for cancer therapy (Jiang et al., 2020). Nevertheless, the treatment outcomes of apoptosis induction is not ideal, owing to apoptosis resistance and immune evasion (Friedmann Angeli et al., 2019). Therefore, there is an urgent need to exploit non-apoptotic cell death to open up new therapeutic avenues.

Ferroptosis, an iron-dependent and reactive oxygen species (ROS) reliant form of RCD which is distinct from apoptosis, necrosis, and other known types of non-apoptotic cell death in terms of morphology, biochemistry and genetics, was discovered and identified by Dixon in 2012 (Dixon et al., 2012). Ferroptosis has attracted extensive attention due to its involvement in neurodegenerative diseases, ischemia-reperfusion injury, cancer, and many other human diseases, which not only provides a novel perspective for us to understand the underlying mechanisms of the disease but also furnishes fresh opportunities for effective treatment (Ooko et al., 2015; Belaidi and Bush, 2016; Ma et al., 2016; Li et al., 2019; Battaglia et al., 2020). Ferroptosis has been found to be strongly correlated to tumor prognosis (Lu et al., 2017; Liu et al., 2020b) and prognostic models based on the ferroptosis-related gene have been successfully established in a variety of cancer, such as hepatocellular carcinoma (Liang et al., 2020), gastric cancer (Jiang et al., 2021), lung cancer (Han et al., 2021; Jin J. et al., 2021), breast cancer (Li et al., 2021) and so on. However, it is not clear whether ferroptosis-related genes are related to the prognosis of patients with melanoma.

**FIGURE 1:**
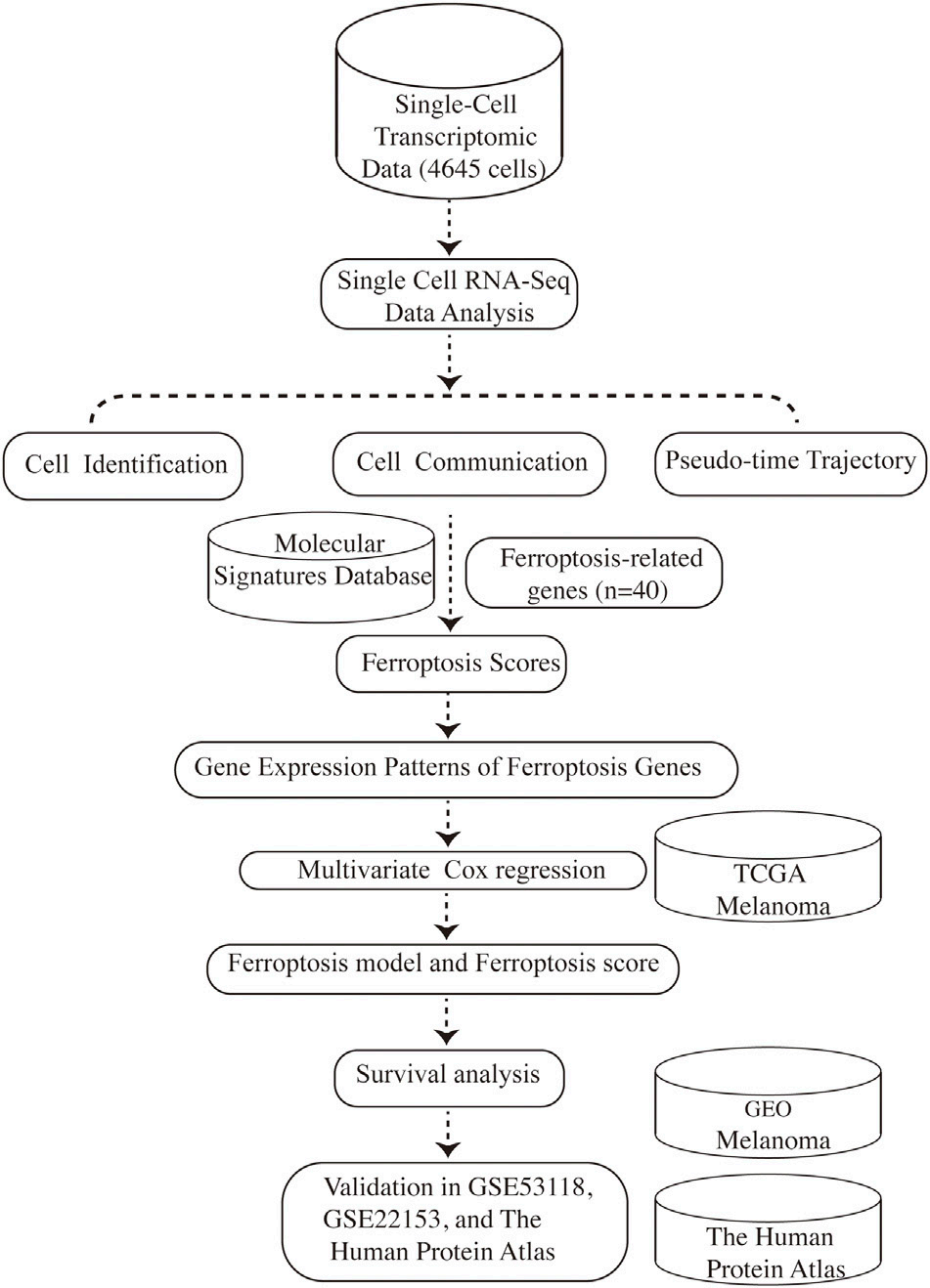
Schematic depicting our study design.

Tumors are highly complex and heterogeneous entities made up of diverse cell populations, as is melanoma (Gonzalez-Silva et al., 2020). Rapid advances in high-throughput sequencing technologies over the years have revolutionized the realm of biology (Stark et al., 2019). The capabilities to study the transcriptome in a more detailed insight using RNA sequencing (RNA-seq) has fueled much groundbreaking discovery and innovation and has become routine in biomedical research (Hong et al., 2020). However, RNA-seq is usually carried out in “bulk”, which represents the average value of gene expression patterns at the whole population level, thus might conceal biologically relevant differences between cells, especially for tumors with complex heterogeneity (Picelli, 2017; Potter, 2018). The advent of single-cell RNA sequencing (scRNA-seq) makes it possible to distinguish gene expression at the single-cell level, which shifts cancer research to a new paradigm and provides stunning new insights into cancer evolution, tumor heterogeneity, and the tumor microenvironment (Hedlund and Deng, 2018; Ren et al., 2018). Herein, for the first time, we take advantage of scRNA-seq data to investigate the connection between ferroptosis-related genes and the prognosis of melanoma, providing new insights for the evaluation and treatment of melanoma. An overview of the research design was presented in Figure 1.

## Materials and Methods

### Data Acquisition

The raw single-cell transcriptome profiling of biopsies from 19 subjects were collected from the supplemental data of the article “Dissecting the multicellular ecosystem of metastatic melanoma by single-cell RNA-seq” published by (Tirosh et al., 2016). RNA-seq data and clinical information of skin cutaneous melanoma (SKCM) patients were retrieved from The Cancer Genome Atlas (TCGA) database (https://tcgadata.nci.nih.gov/), GSE59455 and GSE22153 derived from the Gene Expression Omnibus (GEO) (https://www.ncbi.nlm.nih.gov/geo/).

### Cell Type Identification

Seurat R package (version 4.0.4) was used to analyze the single-cell RNA-seq data (Butler et al., 2018). Using the “NormalizeData” and “FindVariableFeatures” function in Seurat, counts were normalized, and 2000 highly variable genes were then identified. Cells with fewer than 100 genes, more than 10,000 genes, or more than 10% mitochondria content were filtered out (Supplementary Figures S1, S2). Data were scaled and mitochondrial gene expression was regressed out using “ScaleData” function in Seurat using the “vars.to.regress” method. The top 30 principal components and the top 2,000 variable genes were used for the “RunPCA” function in Seurat. Uniform Manifold Approximation and Projection (UMAP) with a resolution of 0.4 was used to show main cell clusters (Becht et al., 2018). To recognize the marker genes, differently expressed genes was performed through the “FindAllMarkers” function in Seurat with wilcoxon rank sum test. Cluster-specific markers were then retained with log2FC > 0.25 (Supplementary Table S1).

### Pathway Activity Calculation

The hallmark gene sets including ferroptosis, hypoxia, oxidative phosphorylation (OXPHOS), and glycolysis were available in the Molecular Signatures Database (MSigDB) (https://www.gsea-msigdb.org/gsea/msigdb/). The major genes in metastasis pathway were then defined and summarized according to the published articles. Subsequently, AUCell package (version 1.8.0) was used to assess individual cells activity for each gene set (Aibar et al., 2017).

### Cell-Cell Communication

To study the communicating interactions between cells and identify the mechanism of the communicating molecules at a single-cell resolution, R package “CellChat” (version 1.0.0) was applied to cells involved in eleven cell groups, including CD8 cells, CD4 cells, B cells, Macrophage/DC cells, EC cells, CAF cells, Malignant cells (Jin S. et al., 2021). Using the “aggregateNet” function in CellChat, the aggregated cell-cell communication network was calculated (Supplementary Figure S3), and the signaling from each cell group was visualized (Supplementary Figure S4). Signaling groups based on their functional or structural similarity were identified *via* the function “computeNetSimilarity” (Supplementary Figures S5, S6). Outgoing or incoming signals of certain cell types were recognized using the function “netAnalysis_signalingRole_heatmap”. Signaling pathways were visualized using the function “netVisual_aggregate”.

### Single-Cell Trajectory Analysis

Monocle2 (version 2.18.0) algorithm was used to calculate pseudo-time, and resulting pseudo-time was scaled from 0 to 1 (Qiu et al., 2017). Indicated channels were used as input dimensions. Then, the hub genes in each cluster were recognized using the function “differentialGeneTest” in monocle2 package (Supplementary Table S2). Top 2000 genes were filtered out based on *q*-values (*q* < 0.01) and used as ordering genes. Expression profiles were reduced to 2 dimensions using the “reduceDimension” function in Monocle 2, *via* the DDRTree method, with max_components = 2. Malignant cells were then ordered using the “orderCells” function and assigned a “pseudotime” value.

Pseudotime-dependent genes (Supplementary Table S3) were determined using the fullModelFormulaStr = “∼sm.ns (Pseudotime)” algorithm included with Monocle 2, *via* the “differentialGeneTest” function.

### Construction and Validation of the Ferroptosis-Related Gene Model

The online Venn diagram analysis (http://bioinformatics.psb.ugent.be/webtools/Venn/) was utilized to discover the intersection genes between ferroptosis and pseudo-time- related genes. Multivariate Cox regression analysis was used in inferring the overlapping genes with the coxph function. The model was verified using TCGA, GSE53118, and GSE22153 dataset. The Human Protein Atlas (HPA, version: 20.1) database (https://www.proteinatlas.org/) was applied to examine the protein expression of the four genes in normal and melanoma tissue (Tang et al., 2017; Uhlén et al., 2015; Uhlen et al., 2017).

### Cell Culture

Normal human epidermal melanocytes (NHEM) were isolated from the foreskin and cultured in MelM-2 (No.2211, ScienCell). The culture medium was supplemented with melanocyte growth supplement and 2.5 ml of fetal bovine serum (FBS). A375, A2058, SK-MEL-28, SK-MEL-5 were grown in Dulbecco’s modified Eagle’s medium (DMEM) supplemented with 10% fetal calf serum and 100 units/ml of penicillin (both obtained from Thermo Fisher, United States). Second- to third-passage melanocytes were transferred into six-well plates.

### Isolation of RNA From Cells and Quantitative Real-Time Polymerase Chain Reaction

RNA extraction kit (ES Science, Shanghai, China) was used for RNA extraction of cells. RNA was quantified and reverse transcribed to generate cDNA. Quantitative realtime polymerase chain reaction (PCR) was performed on SYBR Green fluorescence (TaKaRa, Japan). Fold changes were calculated relative to NHEM by the deltadelta Ct method. The primer sequences are listed in Supplementary Table S4.

### Statistical Analysis

Our analyses were carried out with R software, R version 3.6.3. The corrplot package was used to calculate Spearman’s correlation coefficients (version 0.84). Significance between two groups was estimated by *t* test. Two tailed *p*-value <0.05 were considered statistically significant.

## Results

### Single- Cell Atlas of Cell Subpopulation Profiles Within Melanoma

**FIGURE 2:**
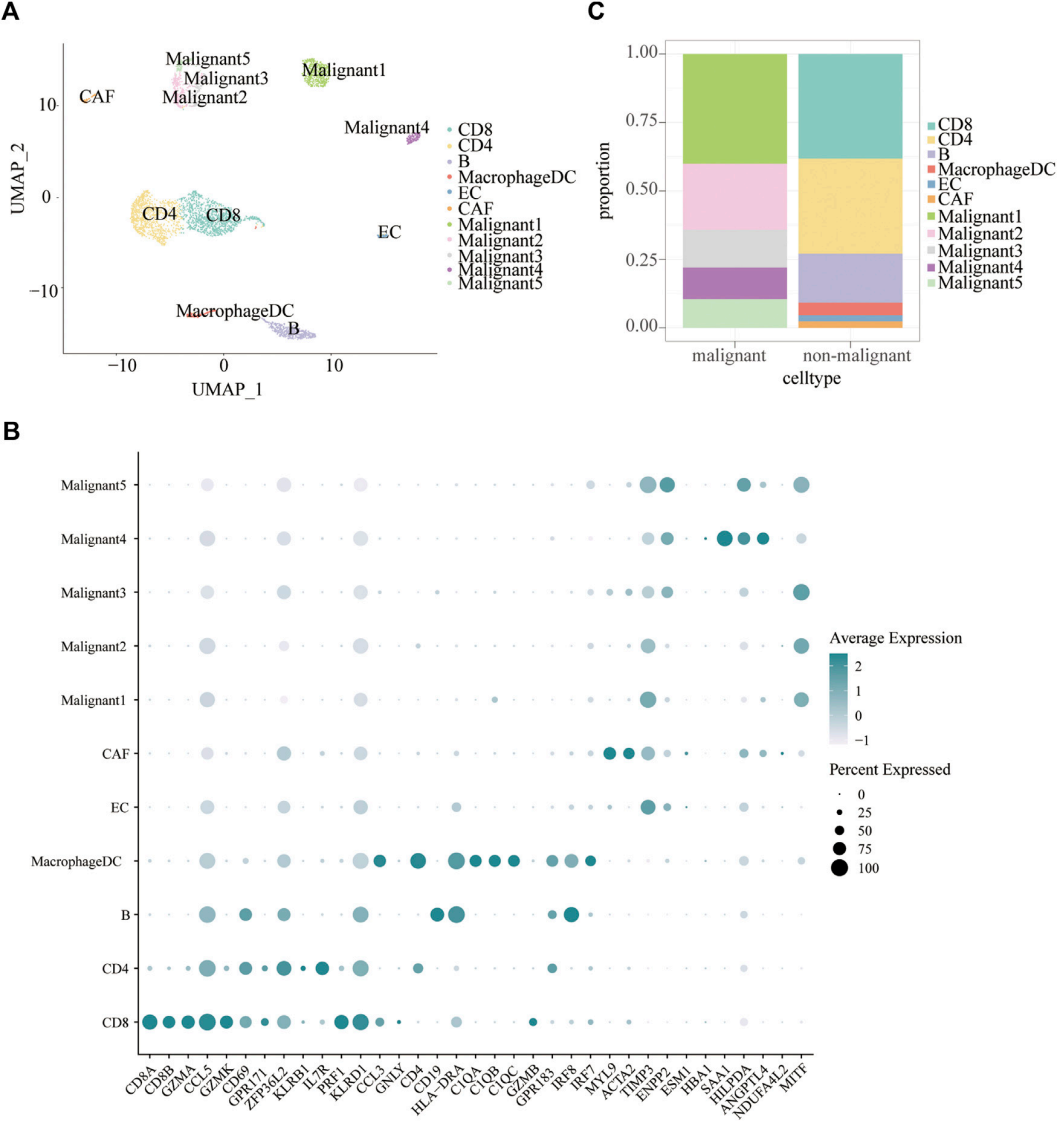
Cell subpopulations in melanoma. **(A)** Uniform manifold approximation and project (UMAP) visualization of transcriptional profiles of melanoma. Each dot represents a single cell, and each color represents a cell type. **(B)** Bar plots showing the proportion of each cell type in malignant and non-malignant cells. **(C)** Dot plot displaying known markers for each cell type.

Tumor heterogeneity is associated with tumor progression, metastasis, and treatment resistance (McGranahan and Swanton, 2017; Gonzalez-Silva et al., 2020). We analyzed single-cell RNA-seq data of 19 melanoma patients to resolve the architecture of the tumor microenvironment. A total of 4,645 cells were analyzed to identify and characterize cell populations. First of all, we reduced the dimensionality of the data by PCA using the top 2000 variable genes. Subsequently, we identified 11 clusters using uniform manifold approximation and projection (UMAP) based on all gene expression levels (Figure 2A). Clusters of non-malignant cells were annotated as CD4 + T cells, CD8+T cells, B cells, macrophages/DC, endothelial cells (ECs), and cancer-associated fibroblasts (CAFs) based on preferentially or uniquely expressed marker genes. Clusters of malignant cells were further divided into five subpopulations. The expression of marker genes on various cell types were shown in Figure 2B. To clearly visualize the cell composition within malignant cells and non-malignant cells, a histogram was made to show the proportion of different cell types in each group subset (Figure 2C). The fraction of cells in each subpopulation revealed that Malignant1 and Malignant2 were the most frequent cell subpopulations in malignant cells while CD4^+^ T cells, CD8^+^ T cells, and B cells were predominant in non-malignant cells (Figure 2C).

### Cell-specific Identification of Ferroptosis-Related Gene Profiles in Melanoma

**FIGURE 3:**
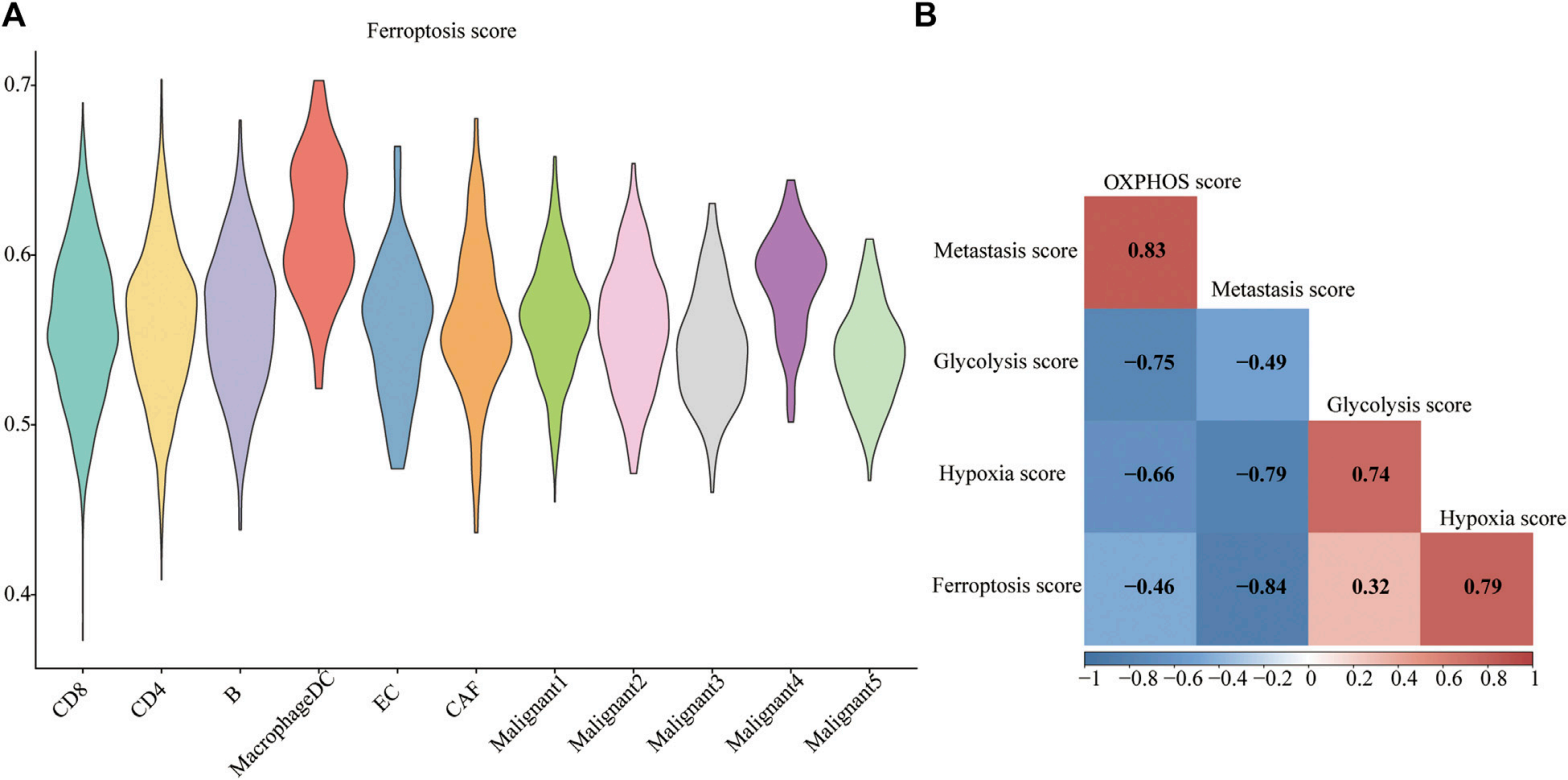
Metabolic landscape for melanoma. **(A)** Distributions of ferroptosis related gene score in each subpopulation. **(B)** Comparing activities of glycolysis, OXPHOS, ferroptosis, hypoxia, metastasis in melanoma.

The ferroptosis-related gene (FRG) (hallmark ferroptosis genes) dataset was downloaded from the Molecular Signatures Database (MSigDB). To examine whether the FRGs are cell-type specific, we focused on the top 40 FRGs. The profile was used to calculate FRG scores in different cell types (Figure 3A). We observed relatively low FGR scores in non-malignant cells compared to malignant cells. Malignant4 ranked first among malignant cells while macrophages/DC took first place among non-malignant cells. Since metabolic reprogramming is a recognized hallmark of cancer, we conducted a series of analyses on metabolic pathways. We investigate correlations among ferroptosis, hypoxia, oxidative phosphorylation (OXPHOS), glycolysis, and metastasis. The correlations between different modules are shown in Figure 3B. A strikingly high negative correlation between ferroptosis and metastasis was observed (Pearson’s *r*: −0.84; Figure 3B). Moreover, a strongly close relationship is depicted between the ferroptosis and hypoxia signatures (Pearson’s *R* = 0.79). OXPHOS activity exhibited a closely correlation with either metastasis (Pearson’s *R* = 0.83) or glycolysis (Pearson’s *R* = −0.75). Indeed, based on this FRG signature, Malignant4 exhibited significantly higher ferroptosis scores than other malignant subtypes, suggesting that functional heterogeneity between identified malignant subtypes may be influenced by ferroptosis status. These results suggested that ferroptosis is cell-type specific in melanoma and related to the prognosis of melanoma.

### Assessment of Cell-Cell Interactions in Melanoma Patients

**FIGURE 4:**
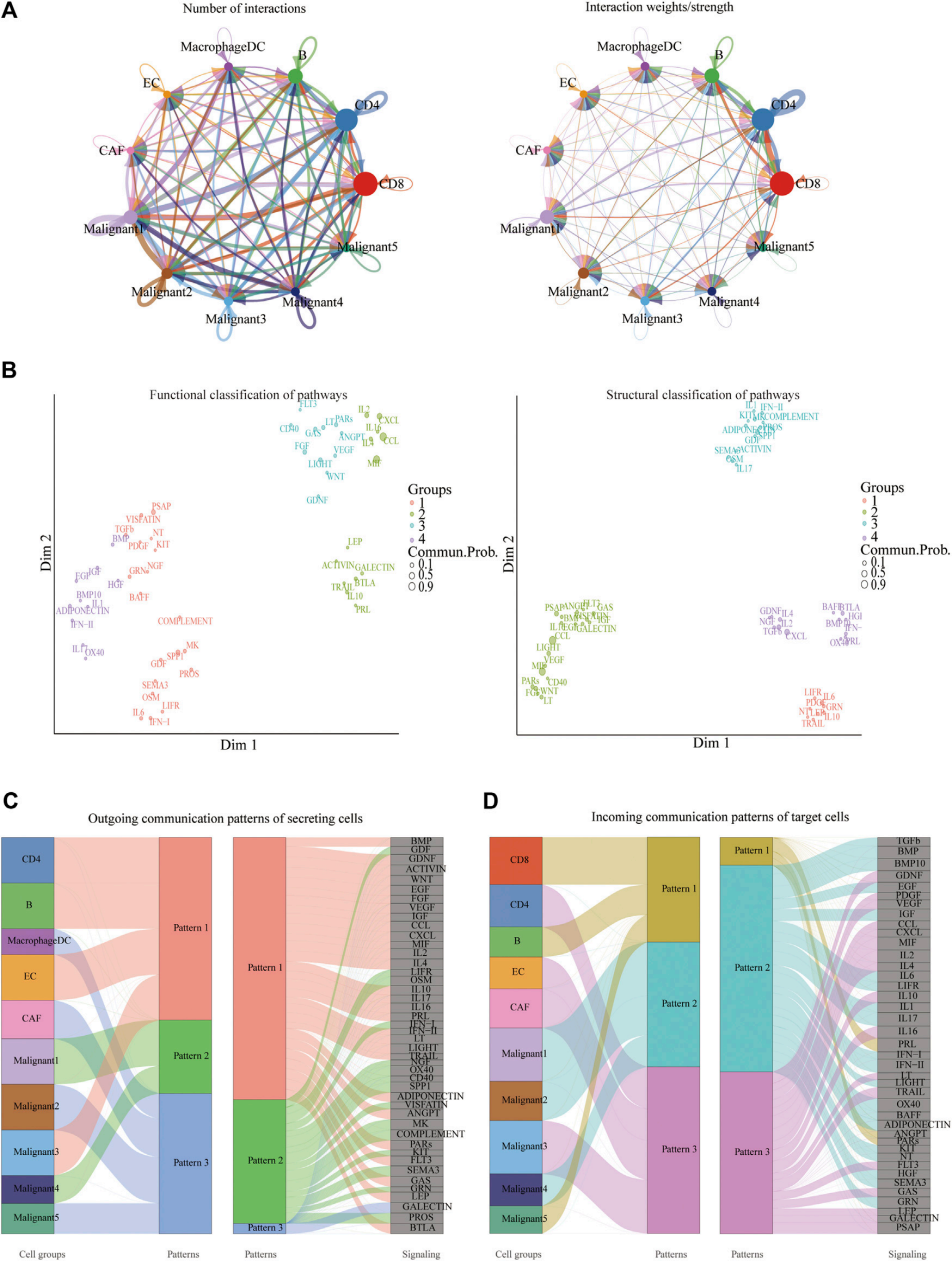
Crosstalk between cancer and immune cells. **(A)** An overview of cell-cell interactions. Arrow and edge color indicate direction. Edge thickness indicates the number (Left) or the weighted (Right) of interaction between populations. The loops indicate autocrine circuits. **(B)** Classification of signaling pathways based on their functional similarity (Left) and structural similarity (Right). Each dot represents a signaling pathway. **(C)** Outgoing signaling patterns of secreting cells. **(D)** Incoming signaling patterns of target cells.

CellChat was used to delineate intricate cell-to-cell communications and predict biologically significant findings from scRNA-seq data. Figure 4A showed the aggregated cell-cell communication network, suggesting the interaction strength and the cell types with significant changes. Different signaling pathways were grouped according to their functional or structural similarity in Figure 4B. Four groups of pathways were identified by functional similarity grouping. Group 1 and Group2 includes inflammatory pathways (e.g., TGFβ, IL, IFN, CCL). Group 3 is dominated by WNT, VEGF, GAS, and CD40 pathways, largely represents signaling between immune cell and malignant cells. Group 4 is composed of EGF, IGF, and HGF pathways, which are promiscuous signals. We are also interested in how multiple cell populations and signaling pathways work together except for the communication of individual pathway. Communication patterns uncovered three outgoing signaling patterns (Figure 4C) and three incoming signaling patterns (Figure 4D).

**FIGURE 5:**
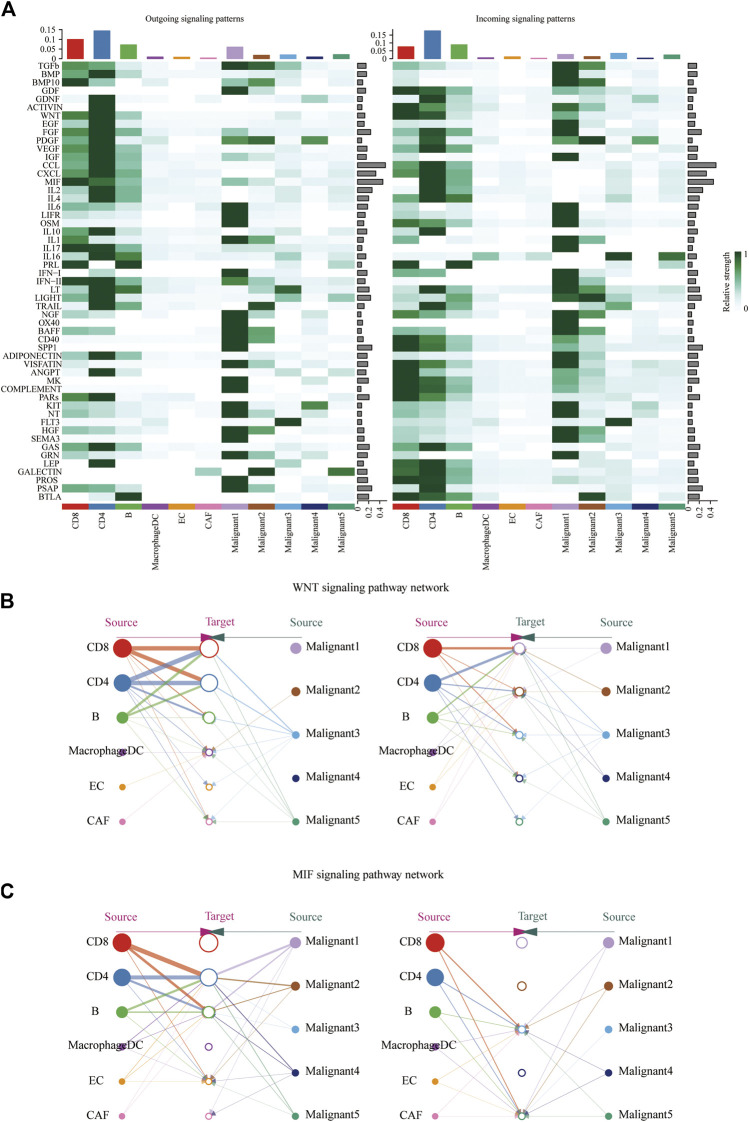
Identification of major signaling changes in melanoma. **(A)** Heatmap shows outgoing (Left) and ingoing (Right) signaling patterns. **(B,C)** Hierarchical diagram shows the inferred cell-cell communication network of WNT signaling **(B)** and MIF signaling **(C)**. Edge thickness indicates the weight of signals between populations.

To further identify differential interactions, a heatmap was performed (Figure 5A). The heatmap uncovered complicated networks of cell-cell interactions between malignant cells and immune cells. Several signaling pathways, including BMP, CXCL, IFNII, IGF, MIF, TGFB, TRAIL, and WNT pathways, were exhibited in Figure 5A. In addition, cross-referencing source and target signaling patterns can provide clues for the autocrine and paracrine pathways in specific cell types. WNT signaling network showed that CD8^+^ T cells were the main source of ligands, which acted in autocrine way between CD8^+^ T cell populations and in paracrine manner from immune cells to malignant3 and malignant5 (Figure 5B). MIF signaling network indicated its largely malignant4- to- CD4^+^ T cells and malignant4-to- CAF signaling, as well as the autocrine-acting pathways between malignant4- to- malignant3 and malignant5 (Figure 5C). Collectively, our results allowed us to gain a deeper understanding of the intricate interactions among cells within the melanoma ecosystem.

### Pseudo-Time Trajectory Revealed Dynamic Change of Malignant Subpopulations and Ferroptosis-Related Gene in Melanoma

**FIGURE 6:**
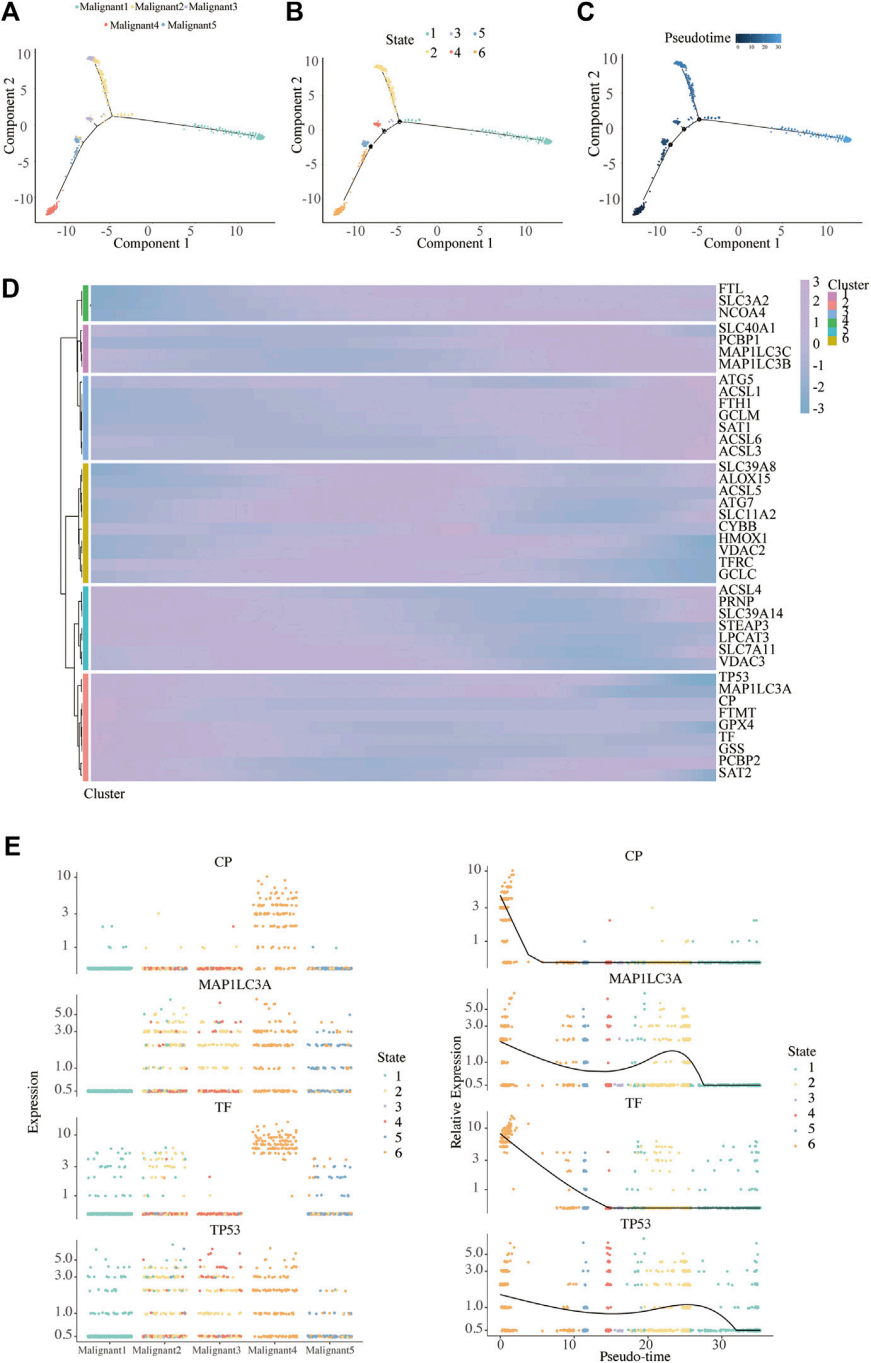
Pseudo-Time analysis. **(A–C)** Distribution of malignant cells on the pseudo-time trajectory. Cells are colored based on malignant subpopulation **(A)**, state **(B)** and pseudo-time **(C)**. **(D)** Heatmap shows the expression of ferroptosis-related genes along the pseudo-time in each cell cluster. **(E)** Clustering and expression kinetics of four genes along the pseudo-time. Dot plot shows the variability of gene expressions in each malignant subpopulation (left) and following pseudotime (right) based on cell states.

We found a major path with three minor branches that can assign pseudo-time- dependent progression states for malignant cells through the single-cell trajectory reconstructed by Monocle (Figures 6A–C). The pseudo-time indicates that Malignant4 is mainly at the start of the projected timeline trajectory, that Malignant5, Malignant3, followed by Malignant 2, is positioned in the middle, and that Malignant1 is at the end (Figure 6A).

We further analyzed the gene expression patterns of ferroptosis genes along the trajectory of melanoma progression. The pseudo-time heatmap of ferroptosis genes showed that their pseudo-time patterns could be divided into six clusters (Figure 6D). The expression of all marker genes in Cluster 1 gradually increased along with pseudo-time while in cluster 3 there was a completely opposite trend. These results expounded the pseudo-time trajectory of malignant subpopulations and the time-dependent changes of FRG in melanoma.

To infer the overlapping genes between ferroptosis and pseudo-time related genes, Venn diagrams were analyzed (Supplementary Figure S7). In particular, four were commonly detected in ferroptosis and pseudo-time related genes, as ceruloplasmin (CP), microtubule associated protein 1 light chain 3 alpha (MAP1LC3A), transferrin (TF), and tumor protein p53 (TP53). We further depicted the dynamic gene expression patterns of CP, MAP1LC3A, TF, and TP53 along the trajectory of melanoma progression and pesudo-time (Figure 6E).

### Establishment and Verification of the Ferroptosis-Related Prognosis Signature in Melanoma

**FIGURE 7:**
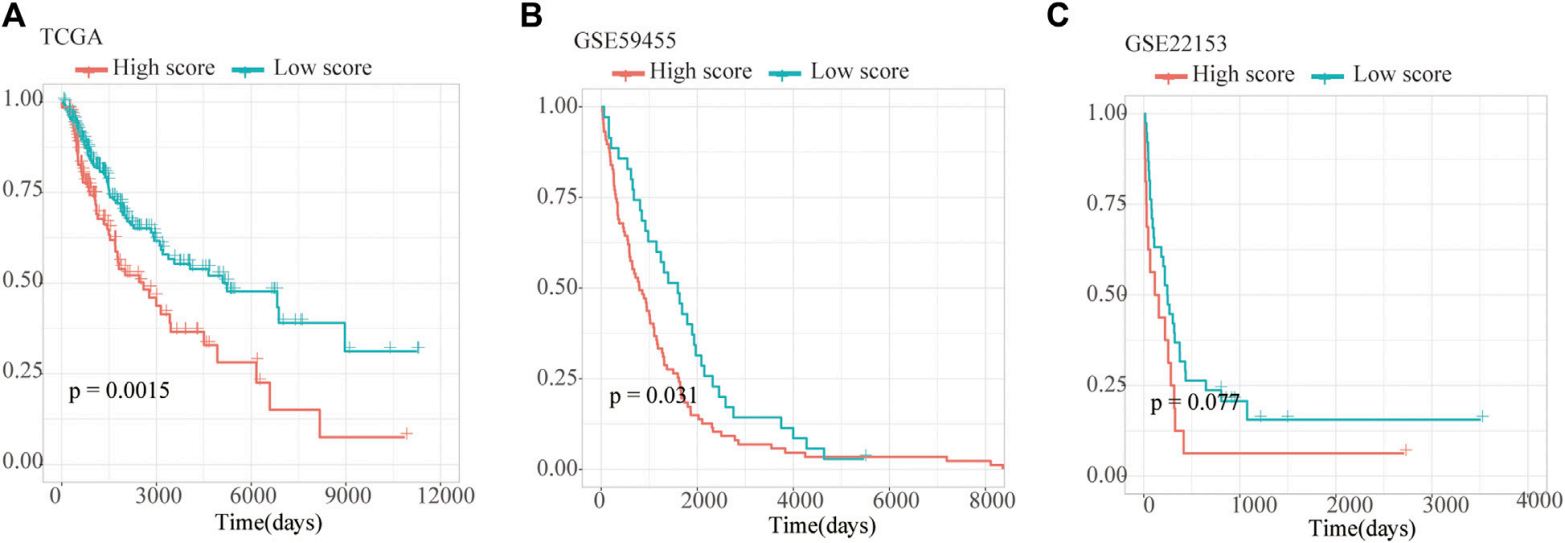
Validation of Ferroptosis prognostic signature. **(A–C)** Kaplan–Meier curves show overall survival of high- and low-score group in TCGA, GSE59455 and GSE22153.

**FIGURE 8:**
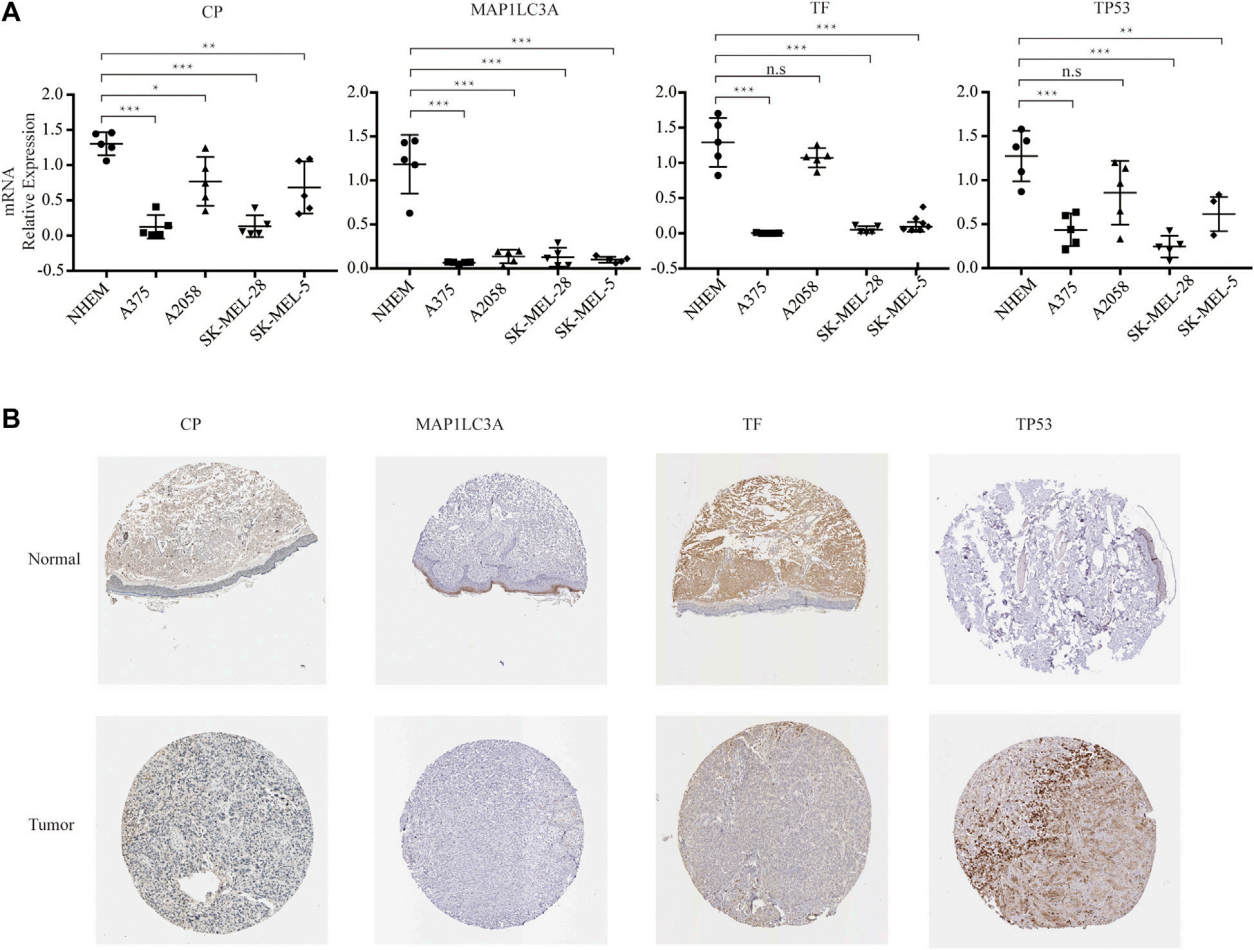
Experimental verification. **(A)** RNA expression of the four genes in multistrain melanoma cell lines and normal human epidermal melanocytes. **(B)** Protein expression of the four genes in normal and melanoma tissue from Human Protein Atlas immunohistochemistry.

As a result, four prognosis-related key FRGs were identified and integrated to construct a prognostic signature for melanoma. The risk score = (−0.09765) ∗ Expression (CP) +(−0.05943) ∗ Expression (MAP1LC3A) + (−0.02367) ∗ Expression (TF) + (0.03474) ∗ Expression (TP53). Cases were divided into high and low ferroptosis risk score groups according to the selected cut-off value 0. Figure 7A indicated that the overall survival outcome of the low-risk score group is better than that of the high-risk score group (*p* = 0.0015). Besides, analysis of COX proportional hazards regression suggested that the signature could serve as an independent predictor of the prognosis of melanoma patients (HR = 2.3293; 95%CI 1.1528–4.706) (*p* < 0.018, Table 1). Kaplan–Meier curve was plotted to evaluate the prognostic ability of the four-gene signature in the external data. Figures 7B,C verified that the overall survival of patients with low risk score was significantly longer than that of patients with high risk score in GSE59455 and GSE22153 dataset (*p* = 0.031, *p* = 0.077). The result of qRT-PCR confirmed that the expression levels of CP and MAP1LC3A were relatively downregulated in A375, A2058, SK-MEL-28, and SK-MEL-5 compared to NHEM. The expression of TF and TP53 were decreased in A375, SK-MEL-28, and SK-MEL-5, while unchanged in A2058 (Figure 8A). To further explore the clinical significance of the signature, we compared the protein expression levels of these 4 genes in normal and melanoma tissues in the Human Protein Atlas database as a reference. The gene expression levels of CP, MAP1LC3A and TF are higher in normal tissue and lower in tumor tissue while TP53 had the reverse tendency (Figure 8B). According to the coefficients of the formula, we inferred that up-regulation of TP53 and down-regulation of CP, MAP1LC3A and TF might have comprehensive effect on increasing the risk of melanomagenesis and poor prognosis.

**TABLE 1:** COX proportional hazards regression analysis.

	Univariate analysis			Multivariate analysis		
	HR	95%CI	*p*	HR	95%CI	*p*
Age (≥58)	1.2482	(0.8799, 1.771)	0.214	1.0758	(0.7487, 1.546)	0.6927
Gender	0.8453	(0.5867, 1.218)	0.367	0.8708	(0.6035, 1.257)	0.4599
TNM Staging	1.516	(1.012, 2.272)	0.0436	1.4274	(0.9432, 2.160)	0.0923
Metastasis	1.296	(0.6045, 2.78)	0.505	1.1452	(0.5306, 2.472)	0.7298
RISK score	2.718	(1.36, 5.435)	0.00467	2.4890	(1.2343, 5.019)	0.0108

HR: hazard ratio; 95% CI: 95% confidence interval.

## Discussion

Melanoma represents the deadliest form of skin malignancies with an increasing incidence worldwide (Schadendorf et al., 2015; Schadendorf et al., 2018). Treatment strategies for advanced melanoma have dramatically changed in the past decade due to the introduction of targeted therapy and immunotherapy (Jenkins and Fisher, 2021). However, despite the application of targeted treatment and immune checkpoint therapies showing a great deal of promise, the development of rapid resistance remains a largely insurmountable challenge (Weiss et al., 2019). Conceivably, a deeper understanding of the complex ecosystem of melanoma is essential to elucidate the treatment targets for melanoma patients. Ferroptosis is a recently coined modality of RCD characterized by excessive accumulation of iron-dependent intracellular ROS production and lipid peroxides (Li et al., 2020). Since the first demonstration in 2012, various cutting-edge research studies have illustrated that ferroptosis took part in various pathological conditions (Han et al., 2020). Studies on ferroptosis in tumors are on the ascendancy (Hassannia et al., 2019; Chen et al., 2021; Shi et al., 2021). In terms of melanoma, the full picture of ferroptosis may be more complex and further investigation is warranted. In the present study, we uncovered a single cell transcriptome profile of melanoma and developed a ferroptosis-related prognostic model.

ScRNA-seq technology has exhibited promising values in exploring the intratumor heterogeneity, cellular differentiation trajectories and intercellular communications at unprecedented resolution level (Lim et al., 2020). Previously established prognostic models of melanoma were based on traditional RNA sequencing (RNA-seq) data, mostly focused on the differences between primary and metastatic tissues, or between normal and tumor samples while ignoring changes in the tumor itself during tumor development (Huang B. et al., 2020; Huang R. et al., 2020). These prognostic models provide valuable insights, but are limited in displaying a comprehensive overview to depict the multi-faceted landscapes and interactome. Herein, for the first time, we used single-cell sequencing data to construct a ferroptosis-related genes and melanoma prognosis model and pay more attention to melanoma itself. We established single cell transcriptome atlas of melanoma to explore the heterogeneity of melanoma more extensively, which is crucial to revealing the therapeutic targets for melanoma patients. First of all, we identified different cell subpopulations within melanoma, and the results showed that there were 11 clusters, which has some discrepancies with the results in previous literature (Tirosh et al., 2016). We performed UMAP to visualize the single-cell RNA-seq data while Tirosh et al. employed another visualization technique t-Distributed Stochastic Neighbor Embedding (t-SNE). The algorithm difference between UMAP and t-SNE leads to the difference in the dimensionality reduction effect. In general, the result of UMAP could better reflect the real global structure with higher stability and less time, while the result of t-SNE has a more compact local structure (Van Der Maaten and Hinton, 2008; McInnes et al., 2018). In summary, UMAP and t-SNE have their own strengths. Our current study utilizes UMAP to better demonstrate the continuity of cell subsets.

In order to further explore the potential cellular cross talk that may contribute to the development and progression of melanoma, we analyzed intercellular communications in detail using scRNA-seq. The complex relationship between malignant cells and immune cells actually shapes the immune tumor microenvironment (TME) of melanoma. The tightly connected communication network uncovered the extensive interactions within melanoma, plenty of which are worthy of thorough studies in the future. BMP signaling plays a pivotal role in the regulation of neural crest and melanocyte programs whether in normal or pathological states (Gramann et al., 2021). It has been reported previously that BMP signaling is active in 80% of melanomas, whereas it is inactive in adult differentiated melanocytes (Gramann et al., 2019; Venkatesan et al., 2018). Recent research has demonstrated that BMP signaling can promote neural crest recognition and accelerate the occurrence of melanoma (Gramann et al., 2021). The activation of BMP signaling can induce Epithelial–mesenchymal transition (EMT), leading to the aggressive phenotype of melanoma (Gao et al., 2021). Ding Wang et al. also reported that ferroptosis inducer erastin significantly regulates the expression of BMPs(Wang et al., 2018), suggesting that ferroptosis may be related with the development of melanoma by regulating the BMP signaling pathway.

WNT-signaling is a highly conserved signaling system, which plays a vital role in the embryonic development and adult life (Keller et al., 2010; Gajos-Michniewicz and Czyz, 2020). Dysregulation of WNT-signaling promotes the initiation and progression of cancer in various ways (Sinnberg et al., 2018). WNT-signaling is so intricate that its exact role in melanoma remains ambiguous. In this study, we identified that CD8^+^ T cells were the main source of ligands in WNT signaling network of melanoma. IFNs type II (*γ*), one of the main cytokines released by effector CD8^+^ T cells, emerges as a crucial component of the tumor immunosurveillance system, which determines the outcome of checkpoint blockade treatment (Dighe et al., 1994; Kaplan et al., 1998; Jorgovanovic et al., 2020). The defect of IFN *γ* signaling pathway is the main resistance mechanism to various checkpoint inhibitors (Hargadon, 2021). IFN *γ* released from immunotherapy-actived CD8^+^ T promotes ferroptosis in tumor cells by decreasing the expression of SLC3A2 and SLC7A11, which is instrumental in boosting the efficacy of cancer immunotherapy (Wang et al., 2019). Thus, IFN *γ* released from CD8^+^ T might be a potential therapeutic target for melanoma treatment through regulating ferroptosis, which needs further follow-up studies to confirm.

Other signaling pathways involved in cell-cell interactions analyzed in this study have showed great importance in the development of melanoma. Tumor necrosis factor-related apoptosis-inducing ligand (TRAIL) signaling have shown anti-tumor effects without harming normal cells (Ashkenazi et al., 1999; Daniels et al., 2005). Innate or acquired resistance to TRAIL signaling frequently occurs in melanoma as many components along the apoptosis pathway can confer resistance to TRAIL (Wu et al., 2021). Macrophage Migratory Inhibition Factor (MIF) is an important immunosuppressive factor (de Azevedo et al., 2020; Yaddanapudi et al., 2013). MIF-CD74 signaling pathway is closely related to immune suppression of innate immunity, thus promoting immune escape and tumor progression (Tanese et al., 2015; Chesney et al., 2017; Gil-Yarom et al., 2017). Furthermore, high MIF expression levels are strongly correlated with poor clinical prognosis in advanced melanoma and was involved in poor responses to anti-CTLA-4 therapy (Ekmekcioglu et al., 2016; Yaddanapudi et al., 2016). When combined with anti-CTLA-4 immune checkpoint blockade, MIF inhibition could overcome resistance to melanoma immune checkpoint blockade therapy by increasing CD8^+^ TIL (de Azevedo et al., 2020), which may release IFN *γ* to induce ferroptosis, thus promoting antitumor responses. Further investigation is nessary to ascertain whether ferroptosis involved in or not, which might be a promosing strategy to enhance the outcomes of melanoma treatment. Collectively, research on the cell-cell communication pathway such as Wnt, MIF, IFN II, TRAIL, and BMP pathways have improved our knowledge of the complicate roles of each pathway in melanoma and verified the reliability of our study. It has also identified a great number of promising targets that can be explored as potential therapeutic interventions in the near future. Obviously, further research is warrant to explore how these signaling pathways interact in melanoma to predict the efficacy of targeted pathway therapy.

An accumulating body of research has outlined the blueprint of ferroptosis as a cancer treatment option (Friedmann Angeli et al., 2019; Su et al., 2020; Ye et al., 2020). Recent studies have revealed that studying ferroptosis in melanoma may provide novel insights into clinical treatments. (Ashrafizadeh et al., 2019; Ubellacker et al., 2020). Wang et al. identified that the CAMKK2‒AMPK‒NRF2 signaling axis can negatively regulate ferroptosis, which might be a promising target to improve the efficacy of immunotherapy and ferroptosis inducer to treat melanoma (Wang et al., 2021). Moreover, Ubellacker et al. reported that lymph can protect metastatic cells from ferroptosis and improve their ability survive during hematologic metastasis (Ubellacker et al., 2020). Previous research exhibited that ferroptosis of melanoma cells could be induced while PD-1/PD-L1 blocking immunotherapy activates tumor-infiltrating CD8^+^ T cells (Lang et al., 2019; Wang et al., 2019), showing that ferroptosis is tightly engaged with the progress of melanoma and the efficacy of existing treatments. However, whether ferroptosis-related genes (FRGs) are related to the prognosis of melanoma needs further elucidated. In order to fill this gap, we established a ferroptosis-related four-gene signature, which included CP, MAP1LC3A, TF and TP53, in melanoma with good prognosis efficacy.

CP is a copper-containing glycoprotein that plays an indispensable role in iron homeostasis. CP prevents ferroptosis induced by erastin and RSL3 through regulating iron metabolism in hepatocellular carcinoma (HCC) cells (Shang et al., 2020). It has been reported that the serum ceruloplasmin level may be a diagnostic indicator for melanoma patients (Ros-Bullón et al., 2001). MAP1LC3A is a driver that promotes ferroptosis according to the note from FerrDb (http://www.zhounan.org/ferrdb/). The expression of variant 1 in MAP1LC3A is commonly silenced at the transcriptional level in numerous cancer cell lines due to epigenetic changes, indicating that it may be implicated in the carcinogenesis of gastric cancer, osteosarcoma, and glioma (Bai et al., 2012; Giatromanolaki et al., 2014; Zhang et al., 2016). Emerging studies have revealed that autophagy plays an essential role in inducing ferroptosis (Hou et al., 2016; Liu J. et al., 2020; Zhou et al., 2020), which suggests that MAP1LC3A, a key molecule in autophagy, might also play an essential role in ferroptosis.

According to the note from FerrDb, TF, and TP53 play a dual role as both a driver that promote ferroptosis and a suppressor that inhibit ferroptosis. TF encodes an iron-transport glycoprotein, which integrates iron into cells through transferrin-receptor (TfR) mediated endocytosis. Transferrin is essential for the induction of ferroptosis and functions as crucial regulators of ferroptosis (Shibata et al., 2021). Transferrin-transferrin receptor (Tf-TfR) complex as a major iron uptake pathway is considered a critical factor causing cancer to be sensitive to ferroptosis inducers (Feng et al., 2020). Recent research revealed the lipogenesis regulator SREBF2 could directly induce the expression of TF which confers the suppression of ferroptosis (Hong et al., 2021). TP53, also known as the “guardian of the genome”, encodes a tumor suppressor protein that plays a vital role in cellular stress (Scatena et al., 2021). Mutations in the TP53 gene occur at a lower frequently in skin cancer compared with all human cancers (Ribeiro Moura Brasil Arnaut et al., 2021; Scatena et al., 2021), indicating the potiential role of TP53 in the induction of ferroptosis. TP53 mutation is one of the most common mutations in UVR-induced DNA damage and leads to tumor initiation and progression (Strashilov and Yordanov, 2021; Torrens-Mas et al., 2020). Although TP53 mutations are not common in melanoma, recent researches have revealed that UVR-induced p53 mutations are tightly associated with the increased incidence of melanoma (Loureiro et al., 2020). TP53 plays the opposite role of “brake” and “accelerate” in regulating ferroptosis in different tumor (Wang eeet al., 2020). On the one hand, TP53 can regulate genes (SLC7A11, SAT1, PTGS2 and GLS2) involved in ferroptosis to suppress tumor development (Yang et al., 2014; Gao et al., 2015; Ou et al., 2016). On the other hand, research shown that p53-p21 transcriptional pathways may negatively regulate ferroptosis in tumor (Tarangelo et al., 2018). After verification with qRT-PCR, we found that TP53 were relatively low in A375, A2058, SK-MEL-25, and SK-MEL-5 compared with NHEM, which might due to the short half-life of wild-type p53. Previous studies have demonstrated that the four genes in our signature are highly involved in ferroptosis in melanoma, which providing theoretical support for the reliability of our prognostic model. Further reserch is necessary to delineate the function and mechanism of these genes in modulating ferroptosis in melanoma, which can provide a novel perspective for the cancer treatment strategies.

Although our research results have certain prospects, there were still some possible limitations. Firstly, we built a prognostic model based on the data collected from the GEO public database, in which the selection bias might be inevitable, but we verified in TCGA database and confirmed the reliability to a certain extent. Besides, the number of samples was limited, and there was a paucity of samples racial diversity. Moreover, Breslow depth and Clark level are essential clinical factors for melanoma prognosis, but the information of them for SKCM patients was not available on the TCGA website. In our study, risk score rather than TNM staging appeared to be statistically significant in the multifactorial COX analyses. This result was somewhat surprising. We speculated the limited sample size and lack of some variables may have a marginally significant impact on outcomes in the Cox model. Second, multicenter, large-scale clinical trials and prospective studies are necessary to validate the clinical applicability of the prognostic model. Last but not least, our study was almost descriptive, and further basic experimental research can promote our understanding of melanoma. Nevertheless, we believe that our study will help understand cellular heterogeneity, intercellular communications, malignant differentiation and ferroptosis in melanoma.

To the best of our knowledge, the present study is the first to explore the expression profile and prognostic values of ferroptosis-related genes (FRG) in melanoma through single-cell transcriptome analysis. In summary, we developed a novel ferroptosis-related prognostic risk model consisting of four encoding gene (CP, MAP1LC3A, TF and TP53) in melanoma, which has significant predictive value. It is our hope that this ferroptosis-related prognostic signature can be used in melanoma patients, which may present valuable information for clinical decision-making and propose novel therapeutic strategies to provide each patient with the best cancer treatment.

## Data Availability

The datasets presented in this study can be found in online repositories. The names of the repository/repositories and accession number(s) can be found in the article/Supplementary Material.
